# Clinical Efficacy and Safety of a Combined Loratadine-Betamethasone Oral Solution in the Treatment of Severe Pediatric Perennial Allergic Rhinitis

**DOI:** 10.1097/WOX.0b013e31819f2105

**Published:** 2009-04-15

**Authors:** Teolinda Mendoza de Morales, Francis Sánchez

**Affiliations:** 1 Instituto de Otorrinolaringologia San Bernardino, Caracas, Venezuela; 2 Policlinica Metropolitana, Caracas, Venezuela

**Keywords:** etamethasone, loratadine, pediatric, perennial allergic rhinitis, quality of life

## Abstract

**Background**

Allergic rhinitis is a highly prevalent condition, particularly among children, whose schoolwork and quality of life may be impaired by its symptoms.

**Methods**

In this prospective, multicenter study, children between 6 and 12 years old with a diagnosis of severe perennial allergic rhinitis received a combination of the nonsedating antihistamine loratadine and the corticosteroid betamethasone in an oral solution for 5 days.

**Results**

The total nasal and ocular symptom score was significantly reduced from 11.4 (± 2.1) before treatment to 2.9 (± 2.4) after treatment (*P *< 0.01). Significant reductions (*P *< 0.01) were also observed for sneezing, nasal pruritus, nasal congestion, rhinorrhea, postnasal drip, and ocular erythema and pruritus. No adverse events were reported, and no subject discontinued treatment.

**Conclusions**

The combination of loratadine and betamethasone in an oral solution was safe and effective as initial short-term treatment for symptoms of severe perennial allergic rhinitis in school-aged children.

## 

Allergic rhinitis (AR) is a widespread health problem in Venezuela, with a reported prevalence as high as 39% [1,2]. Approximately 40% of children and adolescents have AR, although the actual figures may be higher because AR is frequently unrecognized and consequently left untreated [3]. The incidence of AR in children increases with age: Up to 14.9% of 6- and 7-year-old children and as high as 39.7% of 13-and 14-year-old children are affected by AR [4]. The risk of developing AR increases by a factor of 2 if a child has 1 atopic parent and by a factor of 4 if both parents are atopic [5]. Allergic rhinitis tends to be more common in boys than in girls up to the age of 15 years, after which the gender disparity usually ends [6].

School performance and quality of life (QoL) of children with AR are generally worse than those of children without the disorder. Children with AR tend to be more irritable and tired than those without AR and can therefore be inattentive and disruptive in the classroom [4]. In the United States, children are absent for more than 2 million school days annually because of AR. A study of adolescents (N = 1834) in the United Kingdom found that subjects with AR were 40% more likely than those in the control group to drop a grade in English, science, or math in their critically important final General Certificate of Secondary Education examinations, which are administered in the spring when AR symptoms occur, different from practice exams taken in the winter when symptoms were not present [7]. Allergic children are also prone to psychological problems such as low self-esteem, shyness, depression, anxiety, and fearfulness [8-10].

Perennial AR (PAR) is a year-round condition that is generally caused by indoor allergens such as dust mites, mold, cat dander, and cockroach allergens [11,12]. A study of adolescents and adults in the United Kingdom found that 56% of those with AR and 8% of the general population experienced PAR [6]. Perennial AR is associated with at least two of the following symptoms: nasal congestion, sneezing, and serous or seromucus hypersecretion. Congestion and mucus production (postnasal drip) predominate in most patients with PAR; sneezing, nasal pruritus, and aqueous rhinorrhea may be minimal [13]. Many patients with PAR may also be bothered by ocular (itchy, red, or watery eyes) or other non-nasal symptoms, such as headache, thirst, and poor concentration [8,14].

Two widely accepted guidelines recommend the use of a second-generation, nonsedating H_1 _antihistamine in combination with an intranasal corticosteroid (INS) for the treatment of PAR. Guidelines developed by the Allergic Rhinitis and Its Impact on Asthma Workshop recommend this combination as first-line therapy for PAR when congestion is the predominant symptom [15]. The European Academy of Allergology and Clinical Immunology guidelines recommend this combination in children with PAR when neither treatment as monotherapy sufficiently controls symptoms [16].

The nonsedating antihistamine loratadine is a selective H_1_-receptor antagonist with a 24-hour duration of effect and a favorable adverse event (AE) profile [11]. In two double-blind, placebo-controlled, randomized clinical studies with adults and adolescents, loratadine reduced both the nasal and ocular symptoms of PAR and was well-tolerated [17,18]. Loratadine has also been found to reduce nasal and ocular symptoms in children with AR [19,20] and to be safe in children as young as 2 years old [21]. The corticosteroid betamethasone has effectively controlled the nasal symptoms of PAR in a study extending over 28 days. Given its risk for systemic AEs during prolonged treatment, betamethasone is generally used as a short-term treatment for patients with PAR whose symptoms do not respond to other medications [22,23].

This study evaluated the safety and efficacy of an oral solution (1 mg/0.05 mg/1 mL) combining loratadine and betamethasone at doses of 10 mg and 0.5 mg, respectively, as initial, short-term treatment for school-aged children with severe PAR in Venezuela. Severe PAR was defined in this study as PAR that alters nasal airflow, interferes with daily activities, and affects QoL, learning, and sleep. This is the first study of an oral solution of these two agents for the treatment of PAR.

## Methods

This open-label, prospective, multicenter, single-group clinical trial enrolled pediatric subjects presenting to otorhinolaryngology offices of participating clinics for symptoms related to AR. To be included, subjects had to be aged 6 to 12 years and in good general health, with no other relevant clinical conditions. They must also have had a clinical diagnosis of PAR, with the presence of 3 or more of the following symptoms: sneezing, nasal pruritus, nasal congestion, rhinorrhea, postnasal drip, and ocular erythema or pruritus. Perennial AR was confirmed in approximately 40% of subjects with a positive skin prick test reaction or a radioallergosorbent test that was class 2 or higher to house dust mites or cat dander. Further, endoscopy of the upper airways was performed.

The diagnosis of PAR was based on Allergic Rhinitis and Its Impact on Asthma Workshop criteria for moderate to severe persistent AR (symptoms present > 4 days/wk and > 4 weeks/y, with one or more of the following characteristics: troublesome symptoms, sleep disturbance, impairment of daily activities/sports, and reduced productivity in school). Total symptom score for study admission was 8 or more (each symptom scored from 0 [none] to 3 [severe] with a total possible score of 15). Approximately 80% of the subjects reported PAR for > 1 year with symptoms present > 5 days/wk.

Subjects were excluded if they had coexisting chronic conditions (except for asthma, atopic dermatitis, and sinusitis), presence of systemic fungal infection, or cognitive or behavioral disturbance, if they were currently receiving oral or parenteral steroid treatment or concomitant phenobarbital, rifampicin, phenytoin (diphenylhydantoin), or ephedrine, or if they had known hypersensitivity to either loratadine or betamethasone.

The study was conducted according to Good Clinical Practices and was approved by each center's ethics committee. School-aged children who met the inclusion criteria and whose parents signed informed consent forms were admitted to the study (day 0). On days 1 to 5, participants received 10 mL of a loratadine (1 mg/mL)/betamethasone (0.05 mg/mL) oral solution once daily at approximately 8:00 AM. Children were evaluated on days 0 and 6. Individual and total symptom scores (main efficacy parameters) were registered at both visits. Tolerability was assessed by AE reports. No additional medications for the symptoms of AR were permitted during the study.

### Data Analysis

The primary efficacy end point was reduction from baseline in total nasal and ocular symptoms, and individual symptoms (sneezing or nasal pruritus, nasal congestion, rhinorrhea, postnasal drip, or ocular erythema or pruritus). Symptoms were rated on a 4-point scale for severity (0 = none; AEs3 = severe) and frequency (0 = none; 3 = 5 days/wk). Clinical response was evaluated by comparing symptom scores on day 0 and day 6 using the Student *t *test. Each subject served as his or her own control (analysis of difference before and after treatment).

## Results

A total of 100 subjects (69 boys) with a mean age of 9.73 (± 1.77) years were enrolled and included in the analysis (Table 1). Seventy-nine subjects cited pathologic evidence demonstrating that sinusitis (53%) or asthma (44%) was also present. Subjects' additional disorders are listed in Table 2.

**Table 1:** Subject Distribution by Age

Age (yr)	Frequency (n)	%	Accumulated %
6	5	5.0	5.0
7	5	5.0	10.0
8	21	21.0	31.0
9	10	10.0	41.0
10	18	18.0	59.0
11	22	22.0	81.0
12	19	19.0	100.0
Total	100	100.0	--

**Table 2:** Additional Pathologic Conditions in Subject Population at Baseline

Condition	Frequency (n)
Sinusitis	21
Asthma	13
Asthma-sinusitis	8
Asthma-atopic dermatitis	7
Atopic dermatitis	7
Asthma-sinusitis	4
Asthma-atopic dermatitis-sinusitis	3
Asthma-atopic dermatitis-chronic urticaria	2
Sinusitis-cornet hypertrophy, otitis	2
Insect sting	2
Conjunctivitis	1
Epilepsy	1
Asthma-conjunctivitis	1
Sinusitis-passive smoker	1
Atopic dermatitis-passive smoker	1
Sinusitis-nystagmus	1
Sinusitis-conjunctivitis-laryngitis	1

Two patients refused to participate in the study due to concerns about possible AEs; their results were not included.

### Efficacy

**Figure 1:**
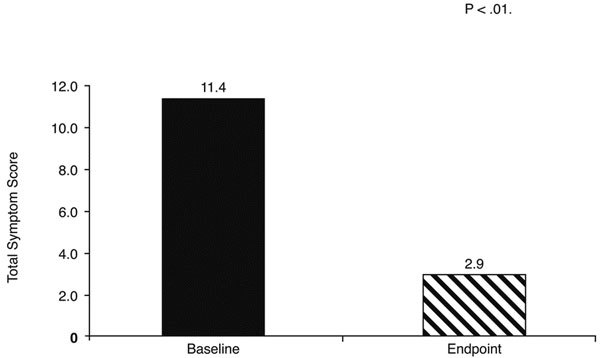
**Total PAR symptom scores before (at baseline) and after 5 days of treatment with loratadine-betamethasone oral solution**.

**Figure 2:**
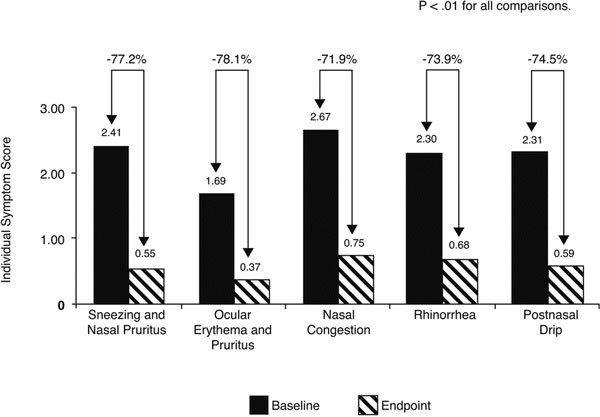
**Individual PAR symptom scores before (at baseline) and after 5 days of treatment with loratadine-betamethasone oral solution**.

The mean total symptom score (nasal and ocular symptoms) decreased from 11.4 ± 2.1 points at baseline to 2.9 ± 2.4 points after 5 days of treatment with the loratadinebetamethasone oral solution (*P *< 0.01) (Figure 1). Significant decreases (*P *< 0.01) from baseline were also reported for each individual symptom (sneezing, nasal pruritus, nasal congestion, rhinorrhea, postnasal drip, and ocular erythema or pruritus), as shown in Figure 2. The greatest improvements in individual symptoms were seen with nasal congestion, with a difference of 1.92 points between the loratadinebetamethasone solution and placebo groups, and with sneezing, with a difference of 1.86 points.

### Adherence

Subjects were instructed to return their medication bottles to the investigators at the completion of the study. All participants reported complete adherence to the treatment regimen.

### Safety and Tolerability

No AEs were reported for any subject, and nobody withdrew from the study.

## Discussion

Allergic rhinitis is a common disease that can be extremely bothersome and, if not treated properly, can lead to significant comorbidities and impairment of patients' daily activities [4,24]. Children can be especially vulnerable to the deleterious effects of AR. Children with AR are prone to comorbid conditions such as asthma, rhinosinusitis, otitis media, and nasal polyposis [4]. They can also fall behind in their schoolwork, become isolated from their classmates, and experience psychological disorders such as depression and anxiety [7-10,25].

The rationale for combining an antihistamine with a corticosteroid is based on each drugs' mechanism of action in both the early and the late phases of allergic reaction. Antihistamines inhibit the release of H_1 _histamine from basophils and mast cells, which alleviates symptoms of sneezing, itching, and rhinorrhea [11,18,26]. By contrast, corticosteroids mitigate nasal blockage (a hallmark of congestion) induced by the chronic inflammatory process and a variety of mediators, which only partially respond to H_1 _antihistamines [11,23,27].

The loratadine-betamethasone oral solution evaluated in this study has the potential to control symptoms in both phases of the allergic reaction. Loratadine exerts an antihistaminic effect that is characterized by attenuation of symptoms resulting from histamine release in the nasal mucosa during the early-phase allergic reaction. Betamethasone has an anti-inflammatory effect, as suggested by the relief of nasal congestion during the late-phase reaction. These results demonstrate that this combination therapy in a liquid formulation provides rapid and continuing relief of nasal and non-nasal symptoms.

The results of this study corroborate those of a previous clinical trial in which loratadine and betamethasone were administered as joint therapy for AR. In this double-blind, parallel-group study in subjects (N = 299) with severe AR and clinically significant obstruction, loratadine and betamethasone were used in combination and individually. The combination was significantly more effective in controlling symptoms, including nasal obstruction, than either drug administered as monotherapy. Further, loratadine, administered with betamethasone, resulted in a significant reduction in the rate of relapse compared with the other treatment regimens [28].

Studies in which loratadine was combined with an INS have demonstrated similar results. Subjects receiving combined adjunctive treatment with loratadine and the INSs mometasone furoate and fluticasone propionate showed significant improvements compared with those receiving placebo in nasal symptoms of AR and QoL [29,30].

An oral formulation, such as the one used in this study, may enhance patient compliance because many children find it difficult to swallow a tablet or use a nasal spray. Two clinical studies of a syrup formulation of loratadine given at doses of 5 mg and 10 mg to children as young as 3 years old found that loratadine reduced both nasal and non-nasal symptoms of seasonal AR and PAR and was well-tolerated [31,32].

Betamethasone was used in the solution at a relatively low dose of 0.5 mg. This is the same dosage used in the loratadine-betamethasone adjunctive treatment study cited above,[28] which was designed to be a study of low-dose betamethasone. As with other first-generation corticosteroids (eg, hydrocortisone, prednisolone, and dexamethasone), betamethasone has been associated with systemic AEs [23]. The use of a lower dose may have contributed to the insignificant AE profile of the loratadine-betamethasone solution. The absence of any AEs in this study also suggests that the solution may be well-tolerated during the repeated short courses needed in the treatment of children with severe PAR.

The interpretation of the results of this study is limited because it was not placebo-controlled; instead, comparison of results was made pre- and posttreatment, with each subject acting as his or her own control. Comparisons of pre- and posttreatment efficacy variables have been utilized in other studies which assessed the clinical impact of corticosteroids in adults and children with AR [33,34]. However, it is possible that, over the course of this study, subjects' symptoms may have abated independent of loratadine/betamethasone treatment as a result of regression to the mean.

Combination loratadine-betamethasone in an oral solution (1 mg/0.05 mg/1 mL) was a safe, well-tolerated, and effective initial, short-term treatment for severe PAR in children between 6 and 12 years old. No AEs were reported for any subject, and nobody withdrew from the study; thus, this formulation may be an appropriate option for short-term control of the symptoms of PAR. Additional investigation of this combination is required to determine its safety profile when used for repeated exacerbations of PAR.

## Note

Supported by Schering-Plough.
